# Supplementary material for: Keep the ball rolling: sexual differences in conglobation behavior of a terrestrial isopod under different degrees of perceived predation pressure
Source: PeerJ. 2023 Dec 19;11:e16696. doi: 10.7717/peerj.16696 (PMC10740659; doi:10.7717/peerj.16696)
Supplement: Supplemental Information 1 — Analyses involving the first trial only, and the effects of treatment regardless of sex. [file peerj-11-16696-s001.docx]

**Keep the ball rolling: sexual differences in conglobation behavior of a terrestrial isopod under different degrees of perceived predation pressure**

Francisco Javier Zamora-Camacho

**Supplementary Material**

**Effect of treatment regardless of sex**

Treatment had a significant effect on unrolling time (mean ± standard error [in s, data prior to log-transformation]; water: 62.425 ± 26.741; rabbit: 102.315 ± 26.741; toad: 159.358 ± 26.741; *F*_2, 126_ = 6.258; *P* = 0.003). The Tukey test detected a significant difference between treatments toad and water (*t*_2, 126_ = 3.524; *P* = 0.002), and non-significant differences between treatments rabbit and water (*t*_2, 126_ = 1.496; *P* = 0.298) and rabbit and toad (*t*_2, 126_ = -2.028; *P* = 0.112).
